# New insights into the magnetism and magnetic structure of LuCrO_3_ perovskite

**DOI:** 10.1107/S2052520624006711

**Published:** 2024-09-03

**Authors:** Angel Muñoz, Javier Gainza, Jian-Shi Zhou, José Luis Martinez, Eva Céspedes, Maria Teresa Fernández-Díaz, José Antonio Alonso

**Affiliations:** a Universidad Carlos III, Avenida Universidad 30, E-28911, Leganés-Madrid, Spain; b Instituto de Ciencia de Materiales de Madrid, CSIC Cantoblanco Madrid E-28049 Spain; c European Synchrotron Radiation Facility (ESRF) 71 Av. des Martyrs 38000Grenoble France; d Materials Science and Engineering Program, Mechanical Engineering, University of Texas at Austin, Austin, TX 78712, USA; e Institut Laue Langevin Grenoble Cedex38042 France

**Keywords:** lutetium chromite, moment canting, G-type magnetic structure, pressure-dependent magnetization, pressure increment of Néel temperature

## Abstract

A polycrystalline sample LuCrO_3_ has been characterized by neutron powder diffraction (NPD) and magnetization measurements. Its crystal structure has been Rietveld refined from NPD data in space group *Pnma*; this perovskite contains strongly tilted CrO_6_ octahedra with extremely bent Cr—O—Cr superexchange angles of ∼142°. The NPD data show that below Néel temperature (*T*_N_ ≃ 131 K), the magnetic structure can be defined as an A-type antiferromagnetic arrangement of Cr^3+^ magnetic moments, aligned along the *b* axis, with a canting along the *c* axis. A noticeable magneto­strictive effect is observed in the unit-cell parameters and volume upon cooling down across *T*_N_. The AC magnetic susceptibility indicates the onset of magnetic ordering below 112.6 K; the magnetization isotherms below *T*_N_ show a nonlinear behaviour that is associated with the described canting of the Cr^3+^ magnetic moments. From the Curie–Weiss law, the effective moment of the Cr^3+^ sublattice is found to be μ_eff_ = 3.55 μ_B_ (calculated 3.7 μ_B_) while the Θ_CW_ parameter yields a value of −155 K, indicating antiferromagnetic interactions. There is a conspicuous increase of *T*_N_ upon the application of external pressure, which must be due to shortening of the Cr—O bond length under compression that increases the orbital overlap integral.

## Abstract 2

The magnetic structure of LuCrO_3_ has G-type antiferromagnetic ordering, with Cr^3+^ moments approximately aligned along the *b* axis, and a subtle canting along **c**. The Néel temperature increases from 112.6 K to 116.0 K upon pressure (up to 1.45 GPa).

## 1. Introduction

The magnetic properties of *R*CrO_3_ (*R* = rare-earth element) (also known as orthochromites) have been thoroughly documented in three successive reviews of perovskite oxides, which collectively encompass a vast array of studies spanning over half a century (Goodenough & Longo, 1970 ▸; Enke *et al.*, 1978 ▸; Endoh *et al.*, 1994 ▸), and have been the topic for many subsequent studies (Bertaut, Bassi *et al.*, 1966 ▸ ▸; Belov *et al.*, 1976 ▸; Hornreich *et al.*, 1976 ▸; Ullrich *et al.*, 1977 ▸; Toyokawa *et al.*, 1979 ▸; Sayetat, 1986 ▸; Courths *et al.*, 1972 ▸; Shamir *et al.*, 1981 ▸; Belik *et al.*, 2012 ▸; Moure *et al.*, 2012 ▸; Weber *et al.*, 2012 ▸; Wang *et al.*, 2019 ▸; Shi *et al.*, 2022 ▸). Additionally, the magnetic structure of orthochromites underwent extensive examination during the growth of neutron diffraction during the 1960s and 1970s. Various magnetic arrangements within the orthorhombic perovskites can be effectively elucidated by referencing the crystal symmetry using the notation pioneered by Bertaut (1963 ▸). Typically, in most cases, the spins are collinearly ordered along the *c* axis of the *Pbnm* cell in orthochromites. However, below the Néel temperature (*T*_N_), the spin structure is influenced by the coupling between the magnetic moment of the rare earth and the spins on Cr^3+^ in certain orthochromites (Hornreich, 1978 ▸).

Even with *T*_N_ of *R*CrO_3_ changing monotonically from Lu to La, irrespective of the rare-earth moment, it appears that the exchange coupling between magnetic *R* and the moment of Cr^3+^ has minimal impact on the Cr—O—Cr coupling. This assumption has been shown to work properly in *R*FeO_3_ (Zhou & Goodenough, 2008 ▸). As in *R*FeO_3_, *R*CrO_3_ oxides are Jahn–Teller inactive, and both families exhibit G-type spin ordering in space group *Pbnm*. Despite sharing the same superexchange coupling parameter *J* in the formula *k*_B_*T*_N_ = 4*S*(*S*+1)*J*, a significant difference between the Néel temperatures of LaFeO_3_ (760 K) and LaCrO_3_ (298 K) can be attributed to the total spin, with *S* = 5/2 for Fe^3+^: *t*^3^*e*^2^ and *S* = 3/2 for Cr^3+^: *t*^3^*e*^0^. However, the dramatic change in *T*_N_ from LaCrO_3_ (320 K) to LuCrO_3_ (140 K) presents challenges in applying the same overlap integral reduction as in *R*FeO_3_. From high-resolution neutron diffraction on all *R*CrO_3_ members, by comparison with structural work on the *R*FeO_3_ family in the literature, some local structural distortions intrinsic to orthorhombic perovskites were identified. The observed variation in *T*_N_ across the *R*CrO_3_ family was well explained only when considering the effect of *t*–*e* hybridization within Cr atoms due to local site distortion and cooperative octahedral-site rotation (Zhou *et al.*, 2010 ▸).

Particularly interesting is the last member of the series, LuCrO_3_, characterized by the most distorted perovskite structure, containing the smallest Cr—O—Cr superexchange angles. Recent studies on the evolution of the unit-cell volume of *R*CrO_3_ oxides unveiled an anomaly occurring for the LuCrO_3_ compound, which has been attributed to the disappearance of the magnetostriction resulting from 3*d*–4*f* couplings (Zhu *et al.*, 2022 ▸). A weak ferromagnetism effect has been described in LuCrO_3_ (Durán *et al.*, 2014 ▸). The magnetic response displays thermal irreversibility between zero-field-cooling and field-cooling conditions which is due to spin canted antiferromagnetic (AF) switching at 116 K. Moreover, a ferroelectric state and multiferroicity has been described in LuCrO_3_ samples below *T*_N_ (Durán *et al.*, 2014 ▸; Preethi Meher *et al.*, 2014 ▸; Alvarez *et al.*, 2014 ▸; Sahu *et al.*, 2008 ▸).

In this paper we give new insights into the magnetic properties and magnetic structure with respect to those described (Bertaut, Bassi *et al.*, 1966 ▸; Durán *et al.*, 2014 ▸; Shamir *et al.*, 1981 ▸). In particular, a spin canting of Cr^3+^ magnetic moments within the considered A-type magnetic arrangement and its thermal evolution below *T*_N_ are described from an NPD experiment, and the evolution of the Néel temperature under pressure (*P* < 1.5 GPa) is reported.

## 2. Experimental

Polycrystalline samples of LuCrO_3_ were prepared by standard solid-state reactions. Mixtures of Lu_2_O_3_ and Cr_2_O_3_ in a stoichiometric ratio were sintered in air at 1253–1723 K with several intermediate grindings. These materials were checked to be single phase using X-ray powder diffraction. NPD patterns were collected at the high-resolution powder diffractometer for thermal neutrons (HRPT) (Fischer *et al.*, 2000 ▸) of the SINQ spallation source at the Paul Scherrer Institute (Villigen, Switzerland). The sample was packed in a cylindrical vanadium holder of 6 mm diameter. A pattern was collected at room temperature using a wavelength of 1.494 Å in a high-intensity mode; the collection time was about 4 h. Low-temperature NPD patterns were sequentially collected at the D20 instrument, a high-flux diffractometer in the ILL (Institut Laue–Langevin, Grenoble, France) reactor with a wavelength of 2.41 Å, in the 1.5–166.2 K temperature interval, with a 5 K step and 15 min collection time for each pattern. The refinement of the crystal and magnetic structures was carried out by the Rietveld method with the *FullProf* software (Rodríguez-Carvajal, 1993 ▸). A pseudo-Voigt function was used to generate the shape of the diffraction peaks. The background was interpolated between areas devoid of reflections. Ultimately, the parameters refined were: scale factor, background coefficients, zero-point error, pseudo-Voigt corrected for asymmetry parameters, positional coordinates, anisotropic displacement factors and occupancy factors. The coherent scattering lengths for Lu, Cr and O, were 7.21, 3.635 and 5.803 fm, respectively.

## 3. Results and discussion

### 3.1. Crystal structure characterization

**Figure 1:**
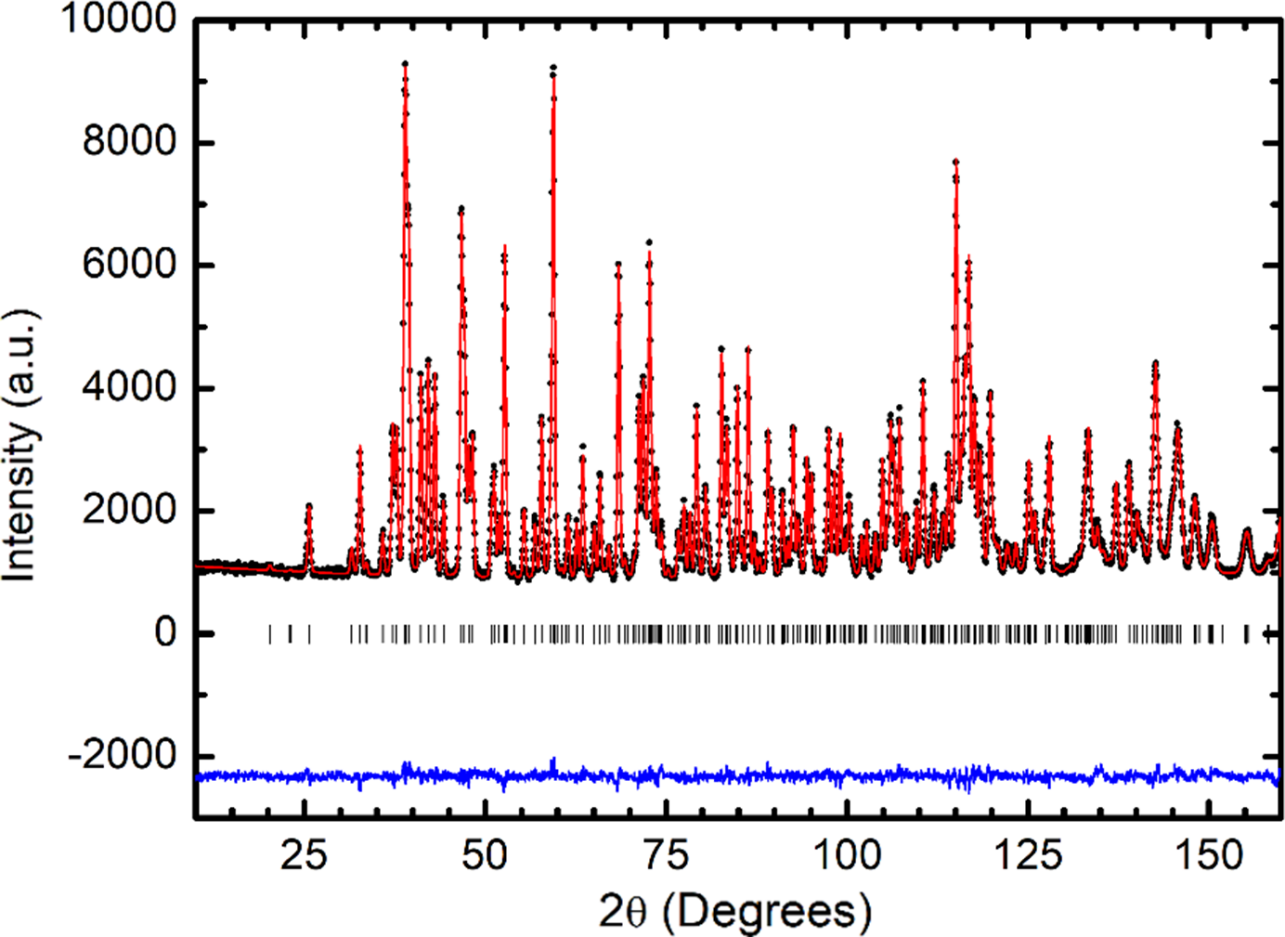
Observed (solid circles), calculated (solid line) and difference (bottom line) NPD patterns after the Rietveld refinement of the crystal structure of LuCrO_3_. The positions of Bragg reflections are represented by a row of vertical lines.

The crystallographic structure of LuCrO_3_ has been refined from a high-resolution powder neutron diffraction pattern collected at room temperature with a wavelength λ = 1.494 Å. The crystal structure was defined in the standard ortho­rhombic space group *Pnma*. The unit-cell parameters, together with the rest of the structural parameters and the conventional discrepancy factors obtained after the refinement are listed in Table 1 ▸. The excellent agreement between the observed and calculated neutron diffraction patterns is shown in Fig. 1 ▸, indicating the quality of the sample; all the Bragg peaks have been indexed and the presence of impurities has not been detected. The most characteristic Cr—O—Cr angles and Cr—O and Lu⋯O distances have also been determined and they are included in Table 2 ▸. LuCrO_3_ is a distorted perovskite with an ortho­rhombic superstructure characterized by *c* < 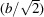 < *a*.

**Figure 2:**
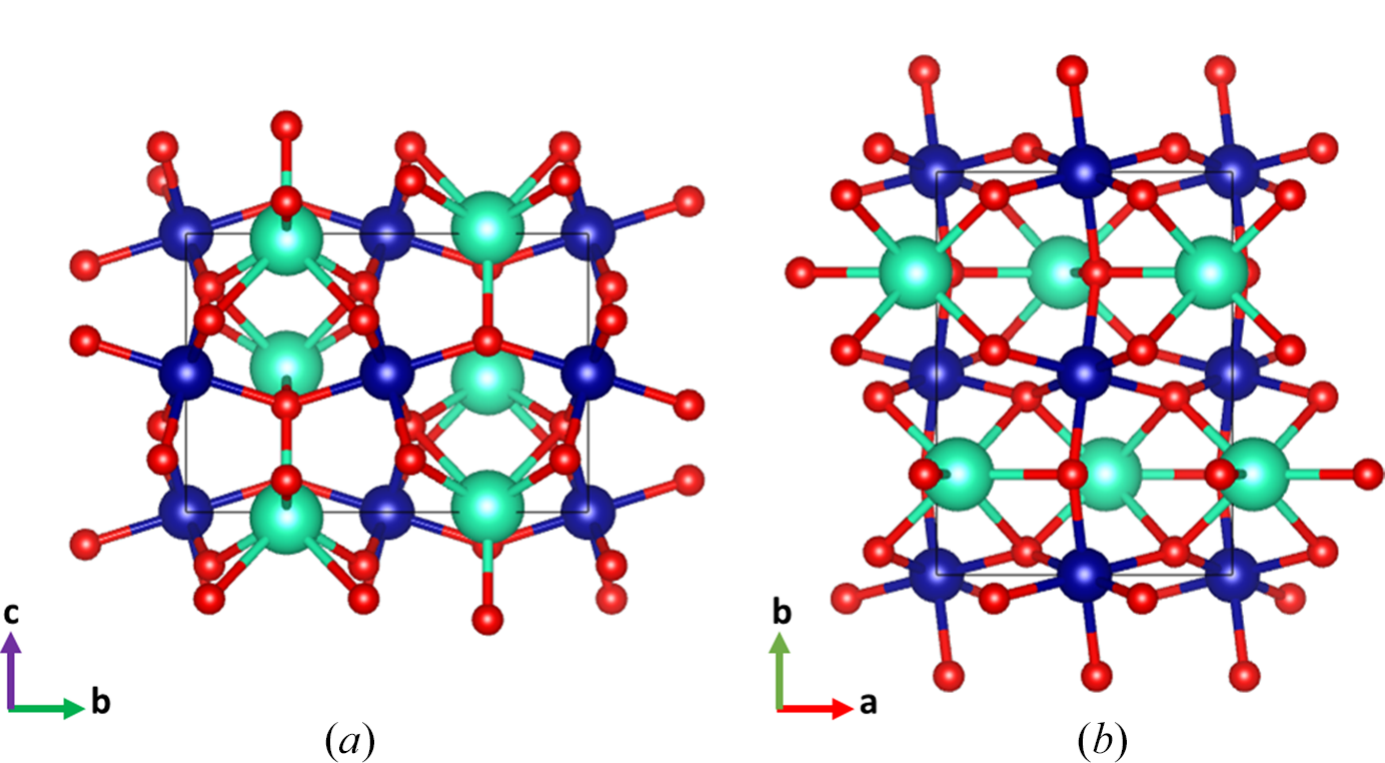
Schematic view of the crystallographic structure of LuCrO_3_ perovskite along **a** (*a*) and **c** (*b*), defined in space group *Pnma*. Green, blue and red spheres represent Lu, Cr and O, respectively.

A schematic view of the crystallographic structure is displayed in Fig. 2 ▸. The crystallographic structure is described by a corner-sharing network of CrO_6_ octahedra with Cr ions at their centre. In the standard *Pnma* setting, the octahedra form chains along the *b* axis linked by the apical O1 (4*c*) oxygen atoms and the equatorial plane of each octahedron lies in the (010) plane. The Cr—O1—Cr and Cr—O2—Cr chains are tilted with respect to the **b** and **c**, respectively. The octahedron rotation can be described by the Glazer notation *a*^+^*b*^−^*b*^−^; as shown in Fig. 2 ▸, the consecutive layers of octahedra are in phase along the **a** direction, whereas along **b** and **c** the layers of octahedra are tilted out of phase. On the other hand, the Cr—O distances in CrO_6_ octahedra span from 1.9766 (3) Å for Cr—O1 to 1.9919 (6) Å for Cr—O2, exhibiting a subtle distortion characteristic of the perovskite superstructures defined in space group *Pnma*. The Lu atom is in eightfold coordination, with distances spanning from 2.1814 (11) Å to 2.6372 (6) Å.

### 3.2. Magnetic characterization

**Figure 3:**
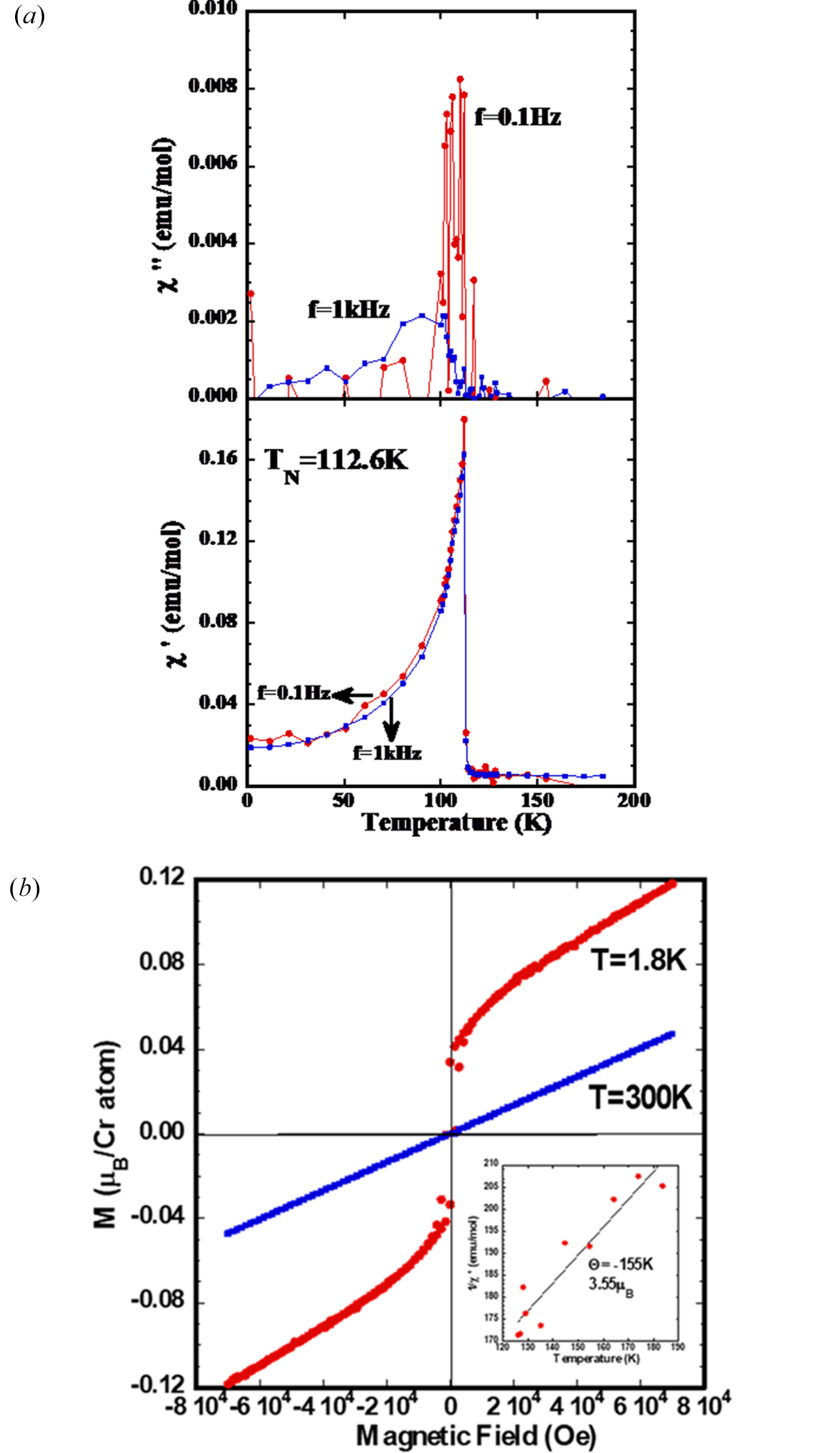
(*a*) AC magnetic susceptibility variation as function of temperature for two different frequencies (0.1 Hz and 1 kHz) for LuCrO_3_. The upper panel shows the imaginary part of χ′′ and the lower panel the real part of χ′. (*b*) The hysteresis cycles for LuCrO_3_ at two different temperatures (1.8 K and 300 K), the inset includes the fitting of the inverse magnetic susceptibility to Curie–Weiss law.

The temperature dependence of the AC magnetic susceptibility (real and imaginary part) is presented in Fig. 3 ▸(*a*). The magnetic susceptibility presents a clear anomaly (cusp like) at *T*_N_ = 112.6 K, that we associate with the antiferromagnetic ordering of the Cr^3+^ ions in LuCrO_3_. This value of *T*_N_ = 112.6 K is lower than that observed by neutron diffraction techniques, probably due to the narrower interval used in the susceptibility measurements (1 K) with respect to neutron data collection (5 K). The imaginary part of the AC magnetic susceptibility also presents a very clear absorption peak at the same temperature (*T*_N_), especially at low frequencies (0.1 Hz). On the other hand, the real part of the AC magnetic susceptibility does not show a significant variation with the applied frequency, at least in the range from 0.1 Hz to 1 kHz.

In a pure antiferromagnetic ordering, we expect a linear behaviour of the ordered magnetic moment in relation to the magnetic field. However, the field dependence of the ordered magnetic moment (below *T*_N_), shows a clear nonlinear behaviour as displayed in Fig. 3 ▸(*b*). This nonlinear behaviour could be associated with a canting of the magnetic moments of the antiferromagnetic structure, as indicated below in the determination of the magnetic structure. This canting moment is ferromagnetic and could be aligned with the external magnetic field applied to the hysteresis loop measurements. This canting moment (ferromagnetic component) is weak, but very clearly observed at low temperature from the extrapolation of the high magnetic field part (4–7 T) giving a value of 0.05 μ_B_/Cr atom at 1.8 K.

Clearly, above *T*_N_ the LuCrO_3_ perovskite behaves as a paramagnetic linear system as indicated in Fig. 3 ▸(*b*) for 300 K. Moreover, from the temperature dependence of the inverse of the AC magnetic susceptibility in the range from 125 K to 200 K, we observed a clear linear behaviour, the slope of which gives rise to a calculated value of the paramagnetic moment of the Cr^3+^ sublattice of 3.55 μ_B_ and Θ_CW_ = −155 K. These data are presented as an inset in Fig. 3 ▸(*b*). The negative sign of Θ_CW_ indicates antiferromagnetic interactions, and the value of 155 K is in the same order of *T*_N_ = 112.6 K. Also, the experimental effective moment of Cr^3+^ (3.55 μ_B_) is close to the spin-only value of Cr^3+^ (3.7 μ_B_) in an octahedral environment.

**Figure 4:**
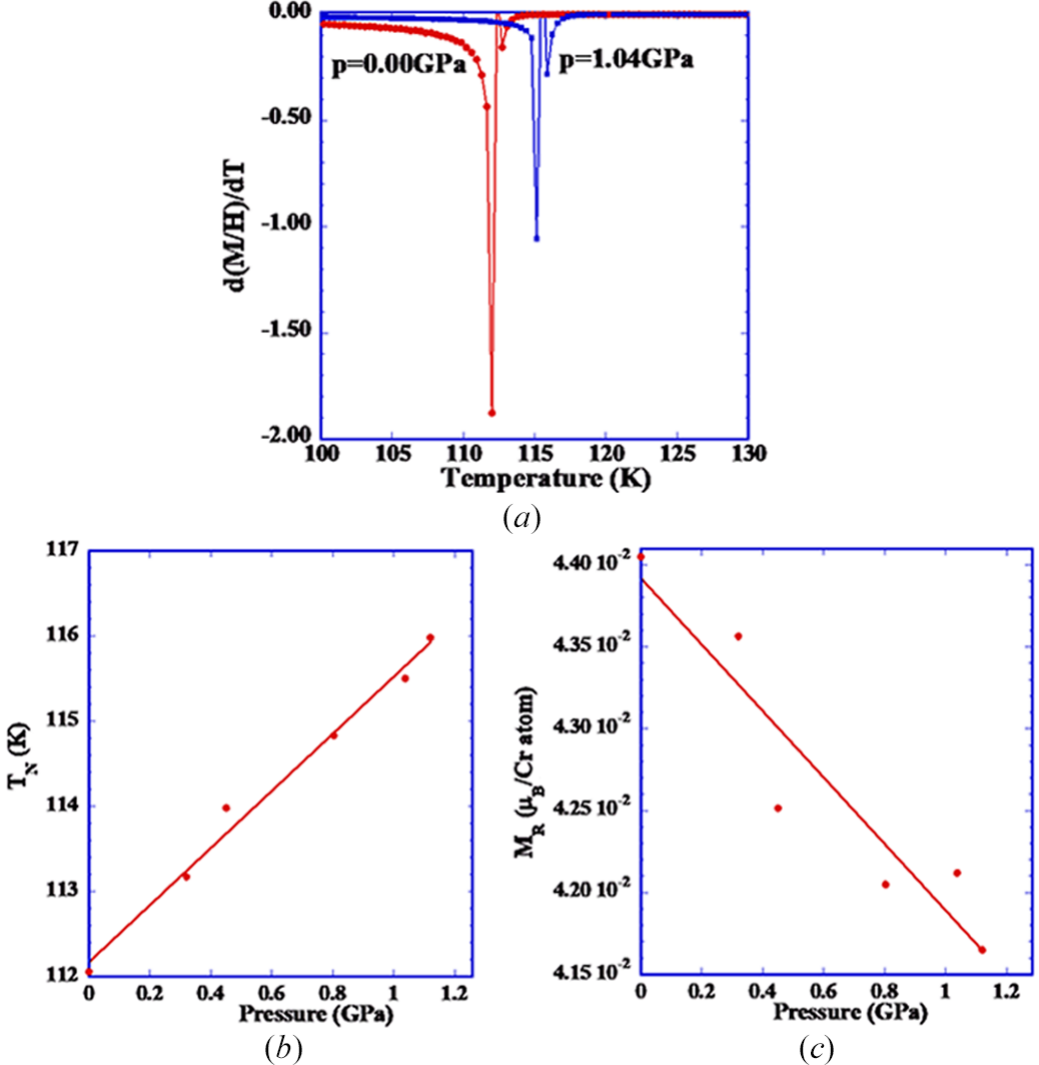
(*a*) Temperature dependence of the magnetic susceptibility for LuCrO_3_ at two hydro­static pressures (0.0 GPa and 1.04 GPa). (*b*) Variation on the antiferromagnetic ordering temperature (*T*_N_) for LuCrO_3_ upon the hydro­static pressure. (*c*) Variation on the remnant magnetization for LuCrO_3_ under hydro­static pressure at a temperature of 5 K.

As indicated in the description of the magnetic (see below) and crystallographic structure, the exchange angles Cr—O1—Cr and Cr—O2—Cr involved in the magnetic superexchange are of the order of 142°, which suggest antiferromagnetic interactions, but still are far from the expected values (180°) for a formal antiferromagnetic interaction. In that case, we could expect that the fairly distorted CrO_6_ octahedra could be very sensitive to the application of an external hydro­static pressure. As a consequence, LuCrO_3_ could have a significant variation of the magnetic properties by the application of moderate hydro­static pressure. Certainly, the bulk magnetizations measurements are not a direct measurement of the change in the exchange angle, but a change in the ordering temperature could be related to the variation of the above-mentioned superexchange paths. Based on this hypothesis, we performed magnetic measurements for LuCrO_3_ in a piston-and cylinder cell, which fits inside the SQUID magnetometer, with a hydro­static pressure range from 0 to 1.4 GPa. The variation of the derivative of the magnetic susceptibility, in a temperature range around *T*_N_, is illustrated in Fig. 4 ▸(*a*) for 0 and 1.04 GPa. The peak in the derivative, associated with the ordering temperature (*T*_N_), changes very significantly in this pressure range. There is an increase of *T*_N_ to higher temperatures, from 112.6 K to 116.0 K, with a total increment of *T*_N_ of 3.4 K at 1.1 GPa.

As a summary, in Fig. 4 ▸(*b*) we present a positive variation of *T*_N_ (defined as a peak in the derivative of the magnetic susceptibility) at two hydro­static pressures. Clearly, the pressure-affected geometry of the CrO_6_ octahedra significantly changes the ordering temperature (*T*_N_). The pressure-induced shortening of the Cr—O bonds leads to an improvement of the orbital overlap and thus the increment of the superexchange interactions and *T*_N_. Zhou *et al.* (2020 ▸) deduced from the unit-cell compression that Cr—O—Cr angles decrease and the tilts of octahedra increase under pressure. The present result suggests that the shortening of Cr—O bond lengths is predominant, with the final effect of increasing the strength of the exchange interactions, giving rise to an increase of the ordering temperature, *T*_N_. A detailed structural/study of fine structure under pressure, involving the determination of the oxygen structural parameters, would be essential to confirm this point.

From the low-temperature (5 K) field-dependence magnetization at different hydro­static pressures, we observe a small variation of the magnetization at high magnetic field (4 T to 7 T), when extrapolated to zero field [see Fig. 4 ▸(*c*)]. This remnant magnetization (MR) varies around 0.25 × 10^−2^ μ_B_ at the maximum pressure of 1.1 GPa. This small variation is inaccurate, as it was extrapolated from a weak pressure dependence.

The external hydro­static pressure clearly modifies the overall magnetic behaviour in LuCrO_3_. Initially the ordering temperature (*T*_N_) increases in a significant way from 112.6 K at atmospheric pressure to 116 K at 1.4 GPa. This overall increase in *T*_N_ is also related to a decrease in the remnant magnetization at 5 K. At 1.4 GPa, the compression of perovskites affects the Cr—O—Cr tilt angles of the octahedra (CrO_6_) and distorts their structure (Xiang *et al.*, 2017 ▸). These data demonstrate the effect of moderate pressure on the bulk magnetic behaviour. However, a more detailed microscopic analysis, from sensitive structural techniques (X-ray or neutrons) will be necessary to determine the details of the different structural parameters under pressure.

#### 3.2.1. Magnetic structure

**Figure 5:**
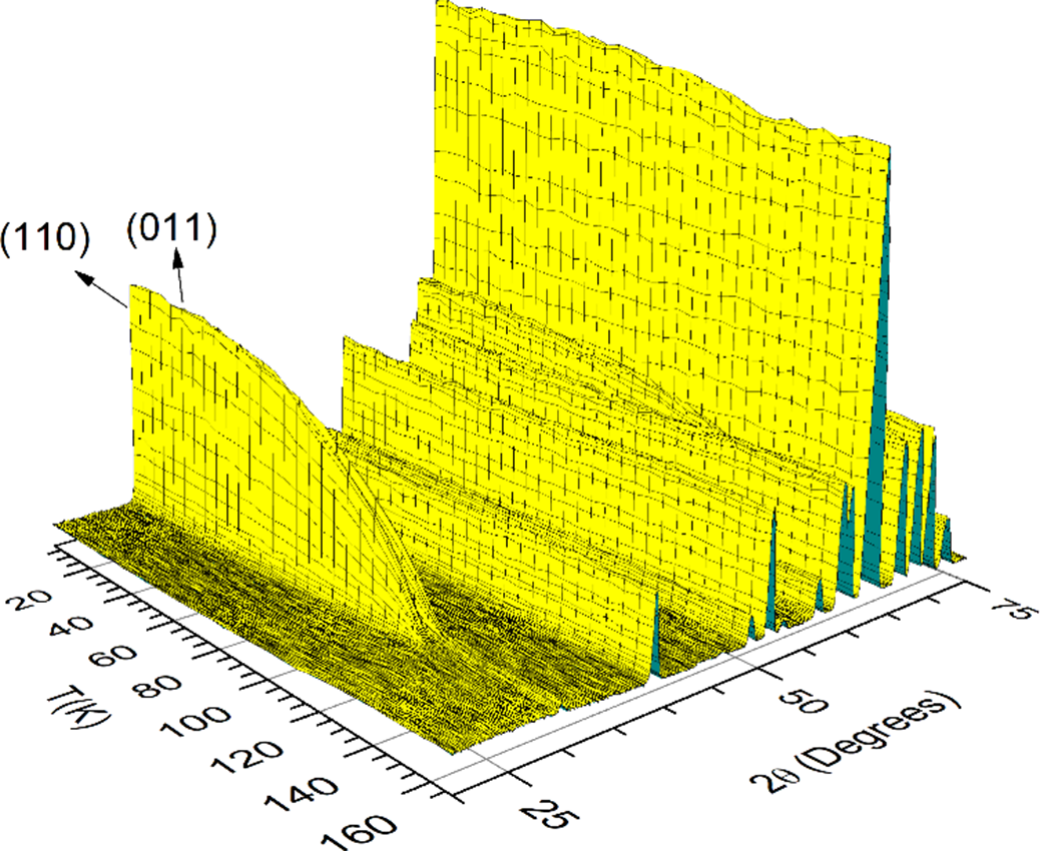
Thermal evolution of the NPD patterns from 1.5 K up to 166.2 K for LuCrO_3_.

In order to determine the magnetic structure and analyse its evolution with temperature a set of NPD patterns were recorded from 1.5 K up to 166.2 K in steps of 5 K, with a wavelength λ = 2.41 Å. The thermal evolution of the neutron diffraction patterns is shown in Fig. 5 ▸. Upon cooling down below 126 K new peaks, such as (110), forbidden in space group *Pnma*, are observed. This indicates the appearance of a magnetic order, characterized by propagation vector **k** = 0. So, the magnetic unit cell coincides with the chemical one. Let us point out that the observed magnetic reflections (*hkl*) seem to accomplish the condition *h* + *l* = 2*n* + 1 and *k* = 2*n* + 1. The magnetic peaks, except for the variation in the intensity, remain stable down to 1.5 K, indicating that no other magnetic transitions occur.

The possible magnetic structures compatible with the crystal symmetry of LuCrO_3_ have been obtained by using the group theory according to the description given by Bertaut (1963 ▸). The basis vectors of the different magnetic structure models have been determined with the *GBasIrep* software integrated in the *FullProf* suite (Rodríguez-Carvajal, 1993 ▸). For the propagation vector **k** = 0, the little group, 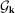, coincides with space group *Pnma*. The different irreducible representations of 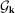, using the Kovalev notation (Kovalev, 1993 ▸) for the symmetry elements, are shown in Table 3 ▸. The basis vectors are given in Table 4 ▸, together with their magnetic space group (Perez-Mato *et al.*, 2015 ▸).

After checking the different solutions, the best one corresponds to the irreducible representation 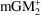, (G_*x*_,A_*y*_,F_*z*_). The description of the magnetic structure, carried out from the NPD pattern at *T* = 1.5 K, are included in Tables 5 ▸ and 6 ▸. The component for m_*x*_ was determined to be zero, which is coherent with the extinction rules for the magnetic mode G_*x*_, that only contributes to the magnetic reflections if *k* = 2*n* and *h*+ *l* = 2*n*+1. The reflections (001), (120) and (021) are not observed and only the G_*x*_ mode can contribute to them.

**Figure 6:**
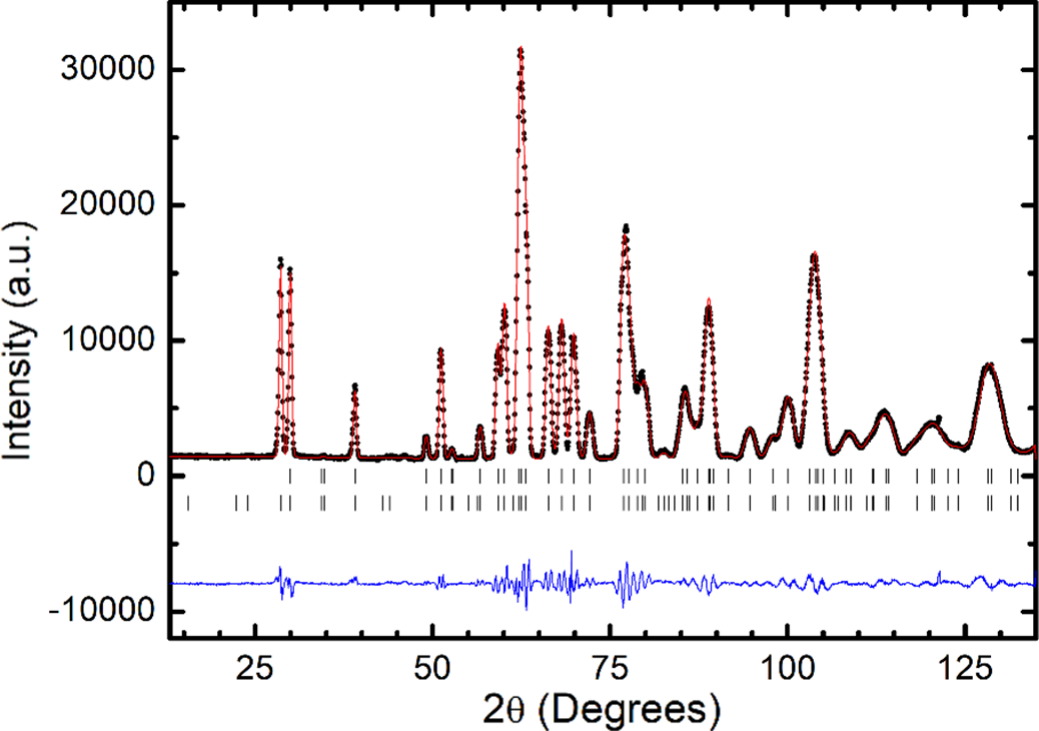
Observed (solid circles), calculated (solid line) and difference (bottom line) NPD patterns at *T* = 1.5 K after the fitting by using the Rietveld profile method. The first row of sticks corresponds to the Bragg nuclear reflections and the second one to the magnetic reflections.

**Figure 7:**
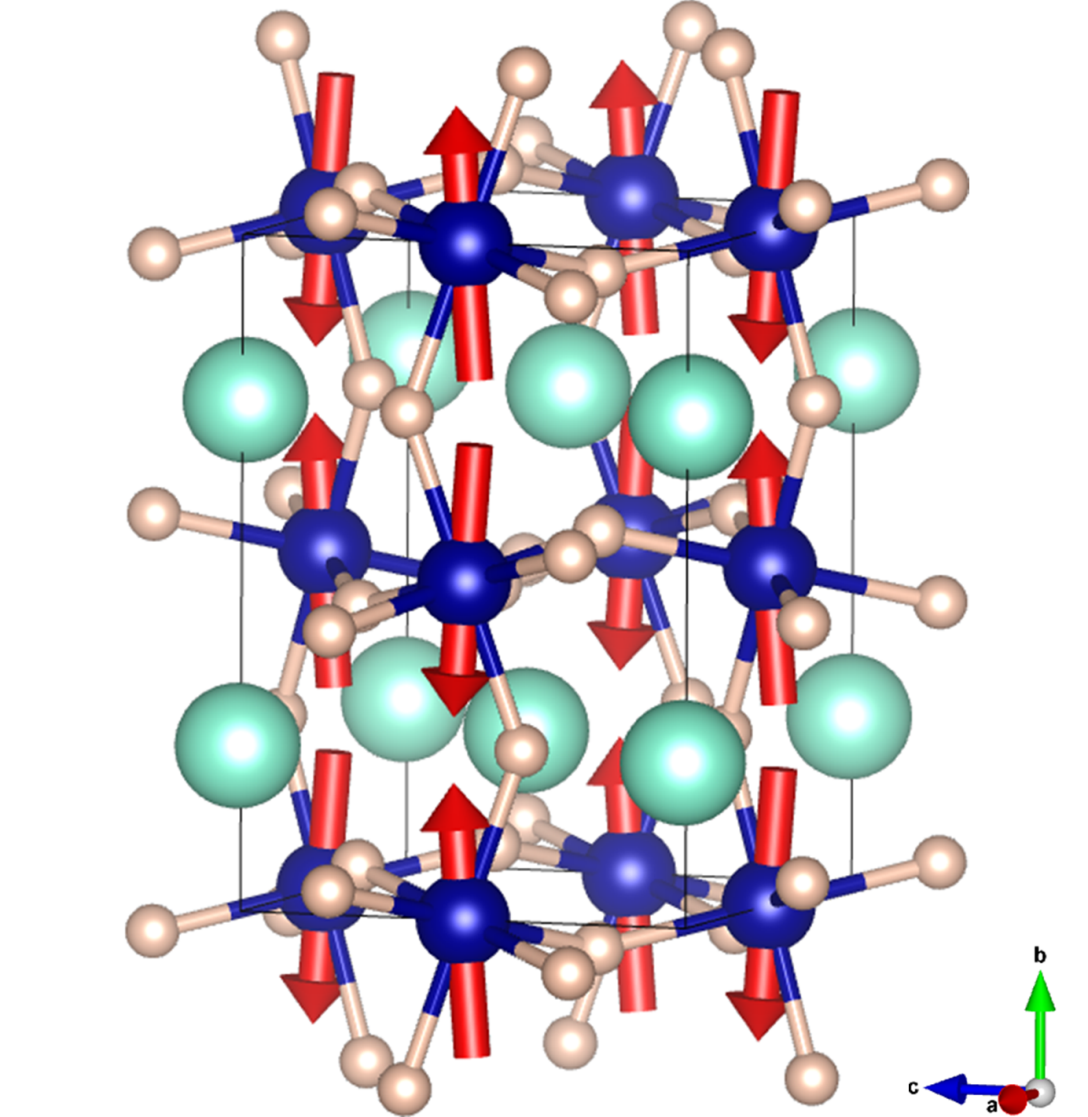
Schematic view of the magnetic structure. The green, blue and pink spheres are the Lu^3+^, Cr^3+^, O^2−^ ions, respectively. The figure highlights the A-type antiferromagnetic arrangement with a subtle canting along the *c* axis.

The good agreement between the observed and calculated patterns is shown in Fig. 6 ▸. A view of the magnetic structure is displayed in Fig. 7 ▸. The magnetic moments have a strong component along **b** and a small component along **c**, which can be described as an A-type antiferromagnetic ordering (*Pnma* setting) with a canting of the moments along the *c* axis. Let us point out that the A-type magnetic structure implies that the magnetic moments coupling is antiferromagnetic both in the (010) plane and between two adjacent planes along **c**. This canting had not been reported before, but it explains the weak ferromagnetism observed in the literature (Hornreich *et al.*, 1976 ▸). The magnetic structure displayed in Fig. 7 ▸ is in contrast with that described before (Shamir *et al.*, 1981 ▸), also obtained by neutron scattering; it is defined as [G_*xz*_63°;—] and this notation implies a G-type coupling with the magnetic moment in the (*xz*) plane (in space group *Pbnm*), but no canting is determined. The work by Hornreich *et al.* (1976 ▸) is in agreement with the pioneering reports by Bertaut, Bassi *et al.* (1966 ▸).

**Figure 8:**
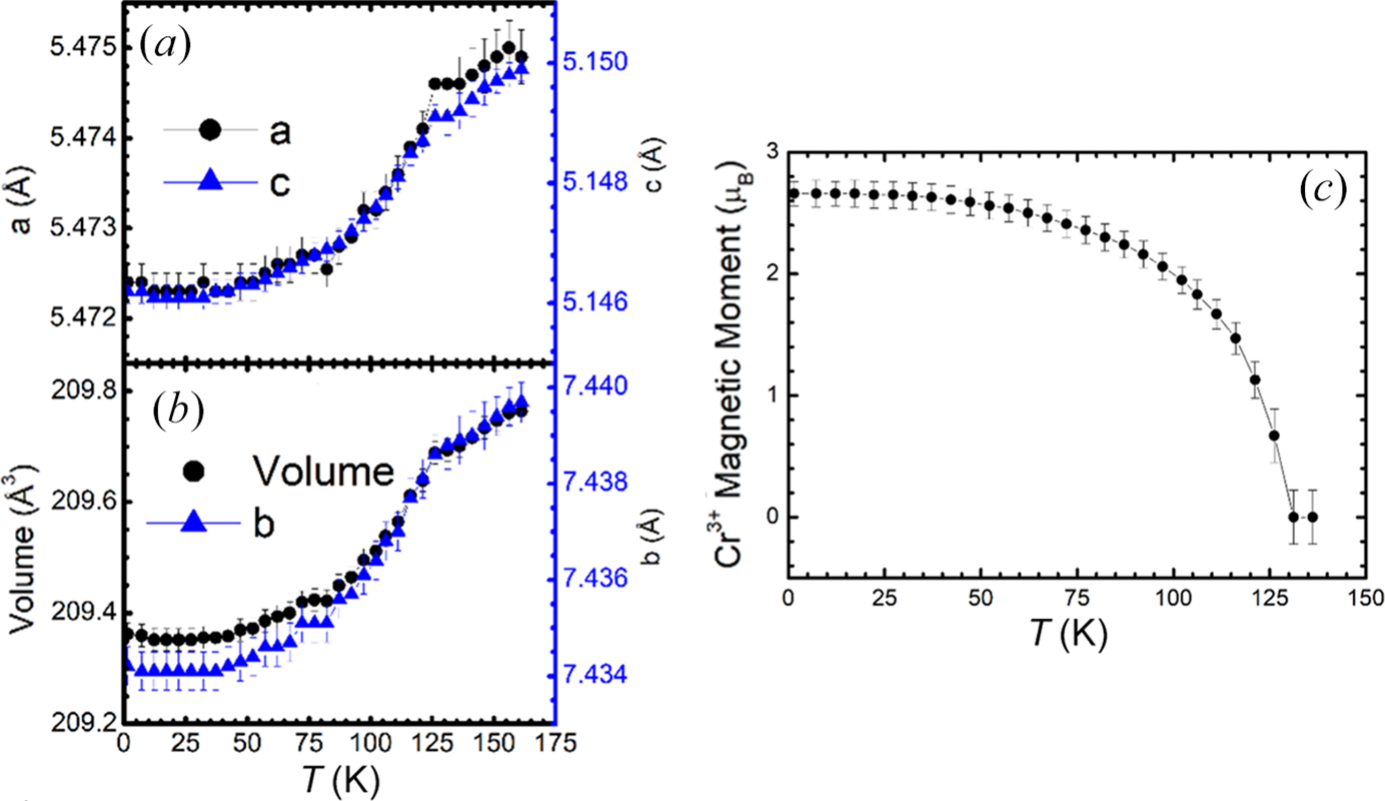
Thermal evolution of (*a*) *a* and *c* unit-cell parameters, (*b*) *b* unit-cell parameter and volume and (*c*) Cr^3+^ ordered magnetic moment.

The thermal evolution of the unit-cell parameters and Cr^3+^ ordered magnetic moments are represented in Fig. 8 ▸. There is a conspicuous magnetostrictive effect that is manifested in an anomaly in the unit-cell parameters and volume upon entering the magnetically ordered phase, just below *T*_N_ ≃ 131 K.

The mechanism that explains the anisotropy in the *A*CrO_3_ perovskites is not clear (Ding *et al.*, 2017 ▸; Bousquet & Cano, 2016 ▸), although it seems to be linked to the size of the *A* cation and also, in the case *A* when is a rare-earth ion with a magnetic moment, to the *f*–*d* exchange between the *A* and Cr sublattices. For the smaller Dy–Lu cations the magnetic moments of the Cr^3+^ cations tend to be orientated towards the *b* axis (Bertaut, Bassi *et al.*, 1966 ▸). The electronic configuration for Cr^3+^ (*d*^3^) is 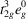, with the two 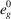 orbitals, 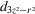 and 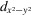, empty. According to the Goodenough–Kanamori rules, in both cases for a 180° *A*—O—*A* bonding the coupling would be antiferromagnetic. In this case the bonding angle for Cr—O1—Cr and Cr—O2—Cr is well below 180°, specifically they are around 142–144° (see Table 2 ▸). The canting along the *c* axis may be related to these significantly bent Cr—O—Cr angles.

## 4. Conclusions

LuCrO_3_ exhibits the well known GdFeO_3_ perovskite superstructure, defined in orthorhombic space group *Pnma*, with narrow Cr—O—Cr superexchange angles due to the small size of Lu^3+^ ions, and stable in the 1.5–300 K temperature range. The magnetic structure, established below *T*_N_ = 126 K from NPD data and 112.6 K from AC susceptibility measurements, is characterized by an A-type antiferromagnetic ordering, with the Cr^3+^ moments approximately aligned along the *b* axis, and a subtle canting along **c**. This canting accounts for the weak ferromagnetism observed in the magnetization isotherms at 1.5 K. *T*_N_ exhibits a clear increase upon the application of an external pressure up to 1.45 GPa, from 112.6 K to 116.0 K. This arises from the shortening of the Cr—O bonds under compression, since previous studies suggest that the tilt angles are enhanced under pressure (Xiang *et al.*, 2017 ▸).

## Figures and Tables

**Table:** 

Space group	*Pnma*
Unit-cell parameters (Å)	*a* = 5.50160 (3), *b* = 7.48093 (3), *c* = 5.17886 (2)
Volume (Å^3^)	213.147 (2)
Wavelength (Å)	1.494
Temperature (K)	295
Discrepancy factors *R*_p_, *R*_wp_, *R*_Bragg_ (%)	2.54, 3.26, 2.30
χ^2^	1.93

**Table:** 

Atom (site)	*x*	*y*	*z*	*B* (Å^2^)
Lu (4*c*)	0.07092 (11)	0.25	−0.01937 (13)	0.184 (10)
Cr (4*b*)	0.0	0.0	0.5	0.09 (2)
O1 (4*c*)	0.45860 (14)	0.25	0.11541 (15)	0.173 (13)
O2 (8*d*)	0.30471 (11)	0.05804 (6)	−0.30993 (10)	0.220 (9)

**Table 2:** Atomic distances (Å) and Cr—O—Cr angles (°) for the closest neighbours obtained after the Rietveld refinement from NPD data at 295 K

Cr—O1 (×2)	1.9766 (3)	Lu⋯O1	2.1814 (11)
Cr—O2 (×2)	1.9799 (6)	Lu⋯O1	2.2442 (10)
Cr—O2 (×2)	1.9919 (6)	Lu⋯O2 (×2)	2.2335 (8)
〈Cr—O〉	1.9827 (2)	Lu⋯O2 (×2)	2.4456 (8)
		Lu⋯O2 (×2)	2.6372 (6)
		〈Lu⋯O〉	2.3823 (3)

Cr—O1—Cr	142.239 (11)	Cr—O2—Cr (×2)	144.06 (2)

**Table 3:** Irreducible representation of the little group 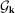 = *Pnma*, with *p* = (½, 0, ½), *q* = (0, ½, 0) and *t* = (½, ½, ½) The notation given by the Bilbao Crystallographic server has been used for the irreducible representations (Aroyo *et al.*, 2006 ▸). *h* are symmetry elements given in Kovalev (1993 ▸) notation.

	*h*_1_	*h*_4_/*p*	*h*_3_/*q*	h_2_/*t*	*h*_25_	*h*_28_/*p*	*h*_27_/*q*	*h*_26_/*t*
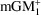	1	1	1	1	1	1	1	1
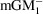	1	1	1	1	−1	−1	−1	−1
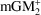	1	1	−1	−1	1	1	−1	−1
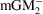	1	1	−1	−1	−1	−1	1	1
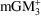	1	−1	−1	1	1	−1	−1	1
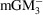	1	−1	−1	1	−1	1	1	−1
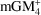	1	−1	1	−1	1	−1	1	−1
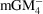	1	−1	1	−1	−1	1	−1	1

**Table 4:** Basis vectors for the Cr atoms, with Cr(1) = (0, 0, ½), Cr(2) = (½, 0, 0), Cr(3) = (0, ½, ½), Cr(4) = (½, ½, 0) For space group *Pnma* and with the atom notation considered before, the basis vectors notations are: **A** = **m**_1_−**m**_2_−**m**_3_+**m**_4_; **C** = **m**_1_+**m**_2_−**m**_3_−**m**_4_; **G** = **m**_1_−**m**_2_+**m**_3_−**m**_4_; **F** = **m**_1_+**m**_2_+**m**_3_+**m**_4_.

	Cr(1)	Cr(2)	Cr(3)	Cr(4)	Magnetic space group	
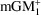	m_*x*_	−m_*x*_	−m_*x*_	m_*x*_	*Pnma*, No. 62.441	
	m_*y*_	−m_*y*_	m_*y*_	−m_*y*_		
	m_*z*_	m_*z*_	−m_*z*_	−m_*z*_		
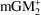	m_*x*_	−m_*x*_	m_*x*_	−m_*x*_	*Pn′m′a*, No. 62.446	
	m_*y*_	−m_*y*_	−m_*y*_	m_*y*_		
	m_*z*_	m_*z*_	m_*z*_	m_*z*_		
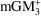	m_*x*_	m_*x*_	m_*x*_	m_*x*_	*Pnm′a′*, No. 62.447	
	m_*y*_	m_*y*_	−m_*y*_	−m_*y*_		
	m_*z*_	−m_*z*_	m_*z*_	−m_*z*_		
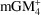	m_*x*_	m_*x*_	−m_*x*_	−m_*x*_	*Pn′ma′*, No. 62.448	
	m_*y*_	m_*y*_	m_*y*_	m_*y*_		
	m_*z*_	−m_*z*_	−m_*z*_	m_*z*_		

**Table 5:** Description of the magnetic structure of LuCrO_3_ under its magnetic space group The discrepancy factors after the refinement of the magnetic structure from NPD data acquired at *T* = 1.5 K with λ = 2.41 Å, are: *R*_Bragg_ = 1.9%, *R*_Bragg Mag_ = 3.5%, χ^2^ = 14.4.

MSG symbol	UNI: *Pn*′*m*′*a*
MSG number	62.446
Transformation to standard setting of MSG	(**a**, **b**, **c**; 0, 0, 0)
Magnetic point group	*m*′*m*′*m* (**a**, **b**, **c**)
Unit-cell parameters (Å, °)	*a* = 5.4724 (2), *b* = 7.4342 (4), *c* = 5.1462 (2)
	α = β = γ = 90
MSG symmetry operations	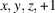
	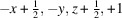
	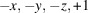
	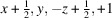
	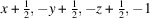
	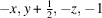
	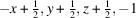
	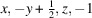
MSG magnetic symmetry centering operations	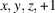
Positions of magnetic atoms	Cr1 Cr 0.00 0.00 0.500
Positions of non-magnetic atoms	Lu1 Lu 0.07135 0.25000 −0.01928
	O1 O 0.45780 0.25000 0.11431
	O2 O 0.30408 0.05801 −0.31035
Magnetic atom, moment components, symmetry constraints and moment amplitude (μ_B_)	Cr1 0.0 2.74 (1) 0.20 (5) (m_*x*_, m_*y*_, m_*z*_) 2.76 (5)

**Table 6:** Complementary information about the magnetic structure of LuCrO_3_ The magnetic structure is given by only one irrep of the parent space group.

Parent space group	*Pnma* (No. 62)
Transformation from parent basis to the one used for the magnetic structure	(**a**, **b**, **c**; 0, 0, 0)
Propagation vector(s)	**k**1 = (0, 0, 0)
Primary irrep(s) label(s) with dimension	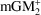 (1)
Description of primary irrep(s)	{1 | 0}: 1
	{2_001_ | ½, 0, ½}: 1
	{2_010_ | 0, ½, 0}: −1
	{2_100_ | ½, ½, ½}: −1
	{−1 | 0}: 1
	{m_001_ | ½, 0, ½}: 1
	{m_010_ | 0, ½, 0}: −1
	{m_100_ | ½, ½, ½}: −1
Secondary irrep(s) label(s)	Not allowed
